# Encephalitis in a Pandemic

**DOI:** 10.3389/fneur.2021.637586

**Published:** 2021-02-10

**Authors:** Dean W. A. Walton, Kiran T. Thakur, Arun Venkatesan, Gerome Breen, Tom Solomon, Benedict Daniel Michael

**Affiliations:** ^1^Department of Neurology, The Walton Centre NHS Foundation Trust, Liverpool, United Kingdom; ^2^Department of Neurology, Columbia University Irving Medical Center and the New York Presbyterian Hospital, New York, NY, United States; ^3^The Johns Hopkins Encephalitis Center, Baltimore, MD, United States; ^4^Department of Social Genetic and Developmental Psychiatry, King's College London, London, United Kingdom; ^5^Clinical Infection Microbiology and Neuroimmunology, Institute of Infection, Veterinary and Ecological Science, Liverpool, United Kingdom; ^6^The NIHR Health Protection Research Unit for Emerging and Zoonotic Infection, Liverpool, United Kingdom

**Keywords:** pandemic, encephalitis, COVID-19, SARS-COV-2, coronavirus

## Introduction

Over a century since the H1N1 influenza pandemic of 1918, we are in the midst of another global pandemic: COVID-19, caused by Severe Acute Respiratory Syndrome- Coronavirus−2 (SARS-CoV-2). While predominantly a respiratory illness, evidence of neurological conditions is arising and we are seeing a plethora of heterogeneous neurology with COVID-19. Analysis of past communicable disease outbreaks and contemporaneous reports will allow us to better understand the potential role of direct neuroinvasion.

## Prior Viral Pandemics and Epidemics

Encephalitis has been observed in previous viral pandemics and epidemics as well as seasonal Coronavirus outbreaks in rare cases. In three cases of encephalopathy with seizures Severe Acute Respiratory Syndrome- Coronavirus-1 (SARS-CoV-1) was identified through brain culture and reverse-transcription polymerase chain reaction (RT-PCR) of CSF (1–3). Three cases of Middle Eastern Respiratory Syndrome (MERS) displayed features of Acute Disseminated Encephalomyelitis (ADEM) and Bickerstaff's Encephalitis but did not find CSF evidence of viral nucleic acid by RT-PCR (4, 5). During the Influenza A pandemic of 2009 (H1N1), cases of Influenza-associated encephalopathy (IAE) increased nearly 7-fold compared to the average over the previous five seasons and seizures and encephalopathy were a common initial presentation among children (6–8). Furthermore, a seasonal coronavirus (OC43) is documented to have caused encephalitis in two immunodeficient children (9, 10). Lastly, although never proven, the 1918 pandemic caused by the H1N1 virus has been associated with the wave of encephalitis lethargica observed at the time (11).

Given this history and these significant findings it is perhaps not surprising that we are seeing a multitude of neurological sequelae associated with the COVID-19 pandemic. However, correlation does not equal causation, and the challenge is distinguishing between neurological complications secondary to critical illness and those directly linked to the virus itself (12).

## Current Evidence of COVID-19 Associated Encephalitis

The encephalitis syndromes seen with COVID-19 are heterogenous in their presentation (13). This undoubtedly represents varied underlying neuropathogenesis. Acute presentations are potentially a consequence of systemic pro-inflammatory cytokines transcending the blood-brain barrier (BBB) or due to direct viral invasion of the central nervous system (CNS) in a small number of cases (12, 14). Later, post-infectious presentations are more likely to be due to immune mediated processes operating through cellular or antibody pathways (6, 15).

Since the first case of COVID-19 encephalitis was reported (16), in whom SARS-CoV-2 RNA was detected in CSF but not on nasopharyngeal RT-PCR, several other case studies have corroborated this phenomenon and demonstrated potential viral invasion as the cause in these cases by positive SARS-CoV-2 RT-PCR in CSF and tissue samples, and evidence of viral particles in neural cell bodies (1, 17, 18). A review article of 21 case reports, found that in 10 patients with proposed encephalitis in whom CSF RT-PCR for SARS-CoV-2 was performed, four were positive and the majority also tested positive for nasopharyngeal RNA (19). The review was critical of attributing symptomology to parenchymal invasion and support for this skepticism also comes from post-mortem data. A study of 43 patients observed marked neuroinflammation in the brainstem of COVID-19 patients at post-mortem with microglia activation and cytotoxic T cell infiltration (20). However, only 21 had evidence of SARS-CoV-2 RNA in post-mortem tissue by PCR and this was not associated with the severity of inflammatory histopathological changes. Moreover, detection of viral RNA in CNS tissue may reflect virus in the blood vessels of cerebral vasculature (as most tissues will have not undergone whole body perfusion via the left ventricle as would be undertaken in murine models) or passive viral entry through a disrupted BBB. Indeed, there is increasing evidence of BBB disruption in COVID-19 (21, 22). In addition to representing an alternative viral entry pathway to the CNS these presentations may represent systemic pro-inflammatory cytokines affecting the CNS, and hence para-infectious, inflammation and maybe also post-infectious antibody response directed against CNS antigens (12).

A further case series of eight patients that examined SARS-CoV-2 antibody (Ab) titres in CSF and serum of COVID-19 patients found detectable SARS-CoV-2 Ab in the CSF of all eight patients but these samples were negative for SARS-CoV-2 by PCR (21). They also demonstrated high CSF titres comparable to serum in four of these patients and evidence of intrathecal synthesis in one patient. This scarcity of detecting SARS-CoV-2 by RT-PCR in CSF of encephalitis cases may reflect disease mechanisms other than direct invasion but alternatively could question the sensitivity of the test itself.

Taken together, these findings highlight the importance of testing the CSF by both PCR and IgG/IgM, and of interpreting antibody findings relative to the concurrent serum titer and to the CSF:serum albumin ratio using Rieber's formula to confirm true intrathecal synthesis. Even prior to COVID-19, many cases of viral encephalitis were well-recognized to be negative in CSF by PCR but antibody positive, such as West Nile virus, and many other flaviviridae and coronaviridea. Nevertheless, in emerging zoonotic infections, such as SARS-CoV-2, in which the pathophysiology is in question, direct visualization of the virus, such as with fluorescent *in-situ* hybridization, remains the gold standard for confirming encephalitis with direct viral neurotropic invasion.

There are now multiple case reports related to post-infectious phenomenon, such as ADEM (17, 23), limbic encephalitis (13, 24), autoimmune encephalitis (25, 26), and in some cases specific autoantibodies directed against CNS antigens have been identified (27).

In the face of amassing evidence of encephalitis in COVID-19, rigorous critique of these case reports and series is needed as several studies lack vital investigations and report diagnoses with minimal evidence (13, 19). However, a common finding among multiple studies is that the presence of neurological complications in COVID-19 has a negative impact on outcomes and delays recovery, although the long-term impact of these complications is not yet known and whether delayed emergent, post-infectious, complications develop is unclear (28–30).

## Moving Forward

As SARS-CoV-2 continues its unrelenting march we will undeniably see further evidence of neurological complications. Currently, while encephalitis cases are sparse it is pertinent that subsequent cases are all identified and meticulously documented in order to classify neurological sequelae in COVID-19. Standardized diagnostic frameworks, such as that proposed by Ellul et al., which utilize the World Health Organization COVID-19 case definitions and apply them to cover neurological clinical syndromes will be valuable for international comparisons of reported cases, case series, and cohort studies (11). In particular, it is important to distinguish between non-specific symptoms associated with critical illness regardless of etiology and those linked with SARS-CoV-2 directly or indirectly (12).

Once classified, consolidating cases and the information they provide is imperative to draw comparisons, appreciate patterns and better understand the neuropathogenesis of COVID-19. Already there are examples of national observational, prospective multi-center, prospective cohort studies (15, 28, 31) that are evaluating the prevalence and outcomes of neurological complications in COVID-19. Efforts to systematically categorize and analyse all relevant publications on a weekly basis is also underway (32). Furthermore, national surveillance programmes that collect and collate neurological cases by allowing clinicians to easily and quickly identify patients in real time are powerful tools for timely appreciation of potential neurological complications of COVID-19; and those which span the clinical neurosciences—including psychiatry and neurosurgery—are of particular value when combating such a rapidly progressing global threat (33).

Lastly, on-going research concerning longer term sequelae and underlying biology is necessary (34–36), especially since the H1N1 pandemic was followed by a wave of encephalitis lethargica that put a considerable strain on health services (37).

## Future Pandemics

While the end of this current pandemic often feels distant, we are hopeful that in the coming months more evidence will be accumulated for potential vaccine candidates. When this pandemic is over, we will need to prepare for future pandemics and epidemics, and ask ourselves- what can we learn from our experience of COVID-19's effects on the brain?

In order to become more proficient and expedient at addressing viral outbreaks we must learn from current events; what has been done well in addition to shortcomings (36). Rapid and early identification of novel zoonoses or mutations is vital to limit spread; containment alone however may prove difficult, as demonstrated by SARS-CoV-2. Once a new threat has reached pandemic proportions, emphasis should turn toward international collaborative efforts of identification, classification, and knowledge sharing, especially with neurological complications where caseloads may be smaller (36, 38).

It is only by implementing a worldwide collaborative response across the neuroscience community that we can tackle such a global disease. Even though this task appears daunting, with the tireless efforts of scientists, medical professionals and researchers what we learn now from this pandemic, even more than in previous pandemics, has the potential for us to be much better prepared for those pandemics yet to come.

## Author Contributions

The final draft was written, reviewed, and approved by all authors.

## Conflict of Interest

The authors declare that the research was conducted in the absence of any commercial or financial relationships that could be construed as a potential conflict of interest.
